# Supplementary material for: Comprehensive deletion landscape of CRISPR-Cas9 identifies minimal RNA-guided DNA-binding modules
Source: Nat Commun. 2021 Sep 27;12:5664. doi: 10.1038/s41467-021-25992-8 (PMC8476515; doi:10.1038/s41467-021-25992-8)
Supplement: Supplementary file 1 — Supplementary Information [file 41467_2021_25992_MOESM1_ESM.pdf]

Supplementary Information for

Comprehensive deletion landscape of CRISPR-Cas9 identifies minimal RNA-guided DNA-binding modules

Arik Shams[1,*], Sean A. Higgins[1,2,3,*], Christof Fellmann[1,4,5], Thomas G. Laughlin[1,6], Benjamin L. Oakes[1,2,3], Rachel Lew[4], Shin Kim[1,2], Maria Lukarska[1,2], Madeline Arnold[1], Brett T. Staahl[1,2,3], Jennifer A. Doudna[1,2,4,7,8,9,10,11], David F. Savage[1,2,^]

Affiliations:
[1]Department of Molecular and Cell Biology, University of California, Berkeley, Berkeley, CA 94720, USA
[2]Innovative Genomics Institute, University of California, Berkeley, Berkeley, CA 94720, USA
[3]Scribe Therapeutics, Alameda, CA 94501, USA
[4]Gladstone Institutes, San Francisco, CA 94158, USA
[5]Department of Cellular and Molecular Pharmacology, University of California, San Francisco, San Francisco, CA 94158, USA
[6]Division of Biological Sciences, University of California, San Diego, San Diego, CA 92093, USA
[7]Graduate Group in Biophysics, University of California, Berkeley, Berkeley, CA 94720, USA
[8]Department of Bioengineering, University of California, Berkeley, Berkeley, CA 94720, USA
[9]Howard Hughes Medical Institute, University of California, Berkeley, Berkeley, CA 94720, USA
[10]Molecular Biophysics and Integrated Bioimaging Division, Lawrence Berkeley National Laboratory, Berkeley, CA 94720, USA
[11]Department of Chemistry, University of California, Berkeley, Berkeley, CA 94720, USA

* These authors contributed equally to this work.
^Correspondence: savage@berkeley.edu.

SUPPLEMENTARY METHODS

Protein expression and purification

A *Streptococcus pyogenes* Cas9 gene containing nuclease-deactivating mutations D10A/H840A (a.k.a. dCas9) was cloned into a pET14b expression vector, encoding a N-terminal 6xHis fusion tag and a C-terminal 2xNLS fusion tag. Specific MISER dCas9 variants were cloned by PCR-amplification (Q5 High-fidelity polymerase, NEB) of the dCas9 gene excluding deleted regions obtained from MISER screen (see Table S4 for primer sequences). Plasmids were verified by Sanger sequencing (UC Berkeley DNA Sequencing Facility). dCas9 and MISER constructs were overexpressed in *E. coli* BL21 (DE3) LOBSTR expression system (Kerafast). Cells were grown in Terrific Broth, modified media with 8 mM $MgCl_2$ and 0.5 glycerol and induced at ~0.6 OD with 0.5 mM IPTG. Cells were resuspended in Lysis Buffer (20 mM HEPES pH 7.5, 1 M KCl, 15 mM imidazole, 1 mM TCEP, 10% glycerol, 0.1 mM PMSF, Roche protease inhibitor tablet), lysed by sonication and clarified by centrifugation, and incubated with Ni-NTA resin to purify soluble fractions. Protein-bound Ni-NTA resin was washed with Wash Buffer (Lysis Buffer + 0.1% Triton X-114), and eluted (Elution Buffer: 20 mM HEPES pH 7.5, 150 mM KCl, 300 mM imidazole, 1 mM TCEP, 10% glycerol). Eluted fractions were subjected to a Heparin Sepharose column (GE Healthcare) for ion-exchange chromatography (300 mM KCl to 1 M gradient), concentrated, and further purified on a gel-filtration column (Superose 6 Increase, GE Healthcare). Protein Storage Buffer was as follows: 20 mM HEPES pH 7.5, 150 mM KCl, 1 mM TCEP, 10% glycerol. Purified protein aliquots were flash-frozen in liquid nitrogen and stored at -80ºC. Concentrations were measured via Nanodrop A280 (ThermoFisher Scientific).

Mammalian cell culture

All mammalian cell cultures were maintained in a 37°C incubator, at 5% CO_2. HEK293T human kidney cells (293FT; Thermo Fisher Scientific, #R70007) were grown in Dulbecco’s Modified Eagle Medium (DMEM; Corning Cellgro, #10-013-CV) supplemented with 10% fetal bovine serum (FBS; Seradigm #1500-500), and 100 Units/ml penicillin and 100 µg/ml streptomycin (100-Pen-Strep; Gibco, #15140-122). U-251 human glioblastoma cells (Sigma-Aldrich, #09063001) and derivatives thereof were cultured in Dulbecco's Modified Eagle Medium/Nutrient Mixture F-12 (DMEM/F12; Gibco, #11320-033) supplemented with 10% FBS and 100-Pen-Strep. U-251 cells were authenticated using short tandem repeat DNA profiling (STR profiling; UC Berkeley Cell Culture/DNA Sequencing facility). STR profiling was carried out by PCR amplification of nine STR loci plus amelogenin (GenePrint 10 System; Promega, #B9510), fragment analysis (3730XL DNA Analyzer; Applied Biosystems), comprehensive data analysis (GeneMapper software; Applied Biosystems), and final verification using supplier databases including American Type Culture Collection (ATCC) and Deutsche Sammlung von Mikroorganismen und Zellkulturen (DSMZ). HEK293T and U-251 cells were tested for absence of mycoplasma contamination (UC Berkeley Cell Culture facility) by fluorescence microscopy of methanol fixed and Hoechst 33258 (Polysciences, #09460) stained samples.

Lentiviral vectors

A set of lentiviral vectors referred to as pSC066-GOI (gene-of-interest) – expressing an EF1a-driven polycistronic cassette containing a MISER-dCas9 or WT-dCas9 KRAB fusion protein, P2A ribosomal skipping element, and a puromycin resistance marker – were based on the pCF525 lentiviral

vector (Watters et al., 2018) derived from pCF204[1]. In brief, the original expression cassette in pCF525 was replaced by the above described EF1a-driven KRAB-MISER-dCas9-P2A-PuroR or KRAB-WT-dCas9-P2A-PuroR polycistronic constructs using custom oligonucleotides (IDT), gBlocks (IDT), standard cloning methods, and Gibson assembly techniques (NEB). Single-guide RNAs (sgRNAs) were expressed from the pCF221 vector[1]. The recipient vector (pCF221-reci) contains Esp3I (BsmBI) restriction sites for sgRNA cloning. The respective sequence (GGAGACGGAGGACGACGAACGTCTCT) is expressed as protospacer in pCF221-reci.

Lentiviral transduction

Lentiviral particles were produced in HEK293T cells using polyethylenimine (PEI; Polysciences, #23966) based transfection of plasmids. HEK293T cells were split to reach a confluency of 70-90% at time of transfection. Lentiviral vectors were co-transfected with the lentiviral packaging plasmid psPAX2 (Addgene, #12260) and the VSV-G envelope plasmid pMD2.G (Addgene, #12259). Transfection reactions were assembled in reduced serum media (Opti-MEM; Gibco, #31985-070). For lentiviral particle production on 10 cm plates, 8 μg lentiviral vector, 4 μg psPAX2 and 2 μg pMD2.G were mixed in 2 ml Opti-MEM, followed by addition of 42 μg PEI. After 20-30 min incubation at room temperature, the transfection reactions were dispersed over the HEK293T cells. Media was changed 12 h post-transfection, and virus harvested at 36-48 h post-transfection. Viral supernatants were filtered using 0.45 μm cellulose acetate or polyethersulfone (PES) membrane filters, diluted in cell culture media if appropriate, and added to target cells. Polybrene (5 μg/ml; Sigma-Aldrich) was supplemented to enhance transduction efficiency, if necessary. Transduced target cell populations (U-251) were usually selected 24-48 h post-transduction using puromycin (InvivoGen, #ant-pr-1; 1.0-2.0 μg/ml).

Mammalian immunoblotting

Stably transduced U-251 cells expressing constructs of interest were washed with ice-cold PBS and scraped from the plates. Cell pellets were lysed in Laemmli buffer (62.5 mM Tris-HCl pH 6.8, 10% glycerol, 2% SDS, 5% 2-mercaptoethanol). Equal amounts of protein were separated on 4-20% Mini-PROTEAN TGX gels (Bio-Rad, #456-1095) and transferred to 0.2 μm PVDF membranes (Bio-Rad, #162-0177). Blots were blocked in 5% milk in TBST 0.1% (TBS + 0.1% Tween 20) for 1 h. All antibodies were incubated in 5% milk in TBST 0.1% at 4°C overnight. Blots were washed in TBST 0.1%. The abundance of β-actin (ACTB) was monitored to ensure equal loading. Immunoblotting was performed using the following antibodies: mouse monoclonal Anti-Flag-M2 (Sigma-Aldrich, #1804, clone M2, 1:500); HRP-conjugated mouse monoclonal Anti-Beta-Actin (Santa Cruz Biotechnology, #sc-47778 HRP, clone C4, 1:250); and HRP-conjugated sheep Anti-Mouse (GE Healthcare Amersham ECL, #NXA931; 1:5000). Blots were exposed using Amersham ECL Western Blotting Detection Reagent (GE Healthcare Amersham ECL, #RPN2209) and imaged using a ChemiDoc MP imaging system (Bio-Rad). Protein ladders were used as molecular weight reference (Bio-Rad, #161-0374).

Cryo-EM image processing

All steps were performed using RELION-v3.1b unless otherwise indicated[2]. Movies were motion-corrected, exposure-filtered, and Fourier cropped to a pixel size of 0.9 Å using and the initial CTF parameters estimated by CTFFIND-v4.1.13[3]. Micrographs were culled by thresholding for CTF-fit resolutions better than 8 Å and manual curation to yield a set of 2554 micrographs used in further processing. An initial set of 97,827 particles were picked using the general model of Boxnet2[4]. These

particles were extract in a 256 pixel box Fourier cropped to 64 pixels (3.6 Å·px^{-1}). Iterative rounds of reference-free 2D classification resulted in 85,327 particles, which were used to generate an ab initio 3D-reference by stochastic gradient descent. Particles were re-extracted and upsampled in a 128 pixel box (1.8 Å·px^{-1}) for further processing. Unsupervised 3D classification did not resolve distinguishable classes. Thus, all particles were subjected to 'gold-standard' 3D auto-refinement using a reference low-pass filtered to 25 Å and a soft shape-mask. This yielded a reconstruction at a nominal resolution of 6.4 Å based on the FSC0.143 criterion and using phase-randomization to correct for masking artifacts[5]. This set of particles was then used to train a picking model with Topaz-v0.2.3[6]. This approach resulted in a set of 288,416 particle coordinates. The new set of particles was extracted in a 128 pixel box (1.8 Å·px^{-1}) and subjected to reference-free 2D classification, which resulted in a set 167,245 particles. Additional attempts at 3D classification did not resolve distinguishable classes. This final set of particles was used for 3D auto-refinement as described above and resulted in a 6.2 Å reconstruction. Further processing using reference-based fitting of particle motion and CTF parameters did not yield improvements. Resolution anisotropy of the final reconstruction was assessed using the 3DFSC web server[7].

Modelling of the cryo-EM map

The previously published coordinate model for the 5.2 Å cryo-EM structure of SpCas9 ternary complex (PDB ID 5Y36) was used as an initial model[8]. To this end, the protein domains were deleted from 5Y36 to match those of Δ4CE. The edited coordinate model was then docked as a rigid-body into the RELION post-processed map using ChimeraX-v1.1, which resulted in a cross-correlation value of 0.73 against a 6.2 Å map simulated from the coordinate model[9]. For display purposes, a denoised version of the Δ4CE map was generated with LAFTER as part of the CCPEM-v1.4.1 suite[10].

SUPPLEMENTARY FIGURES

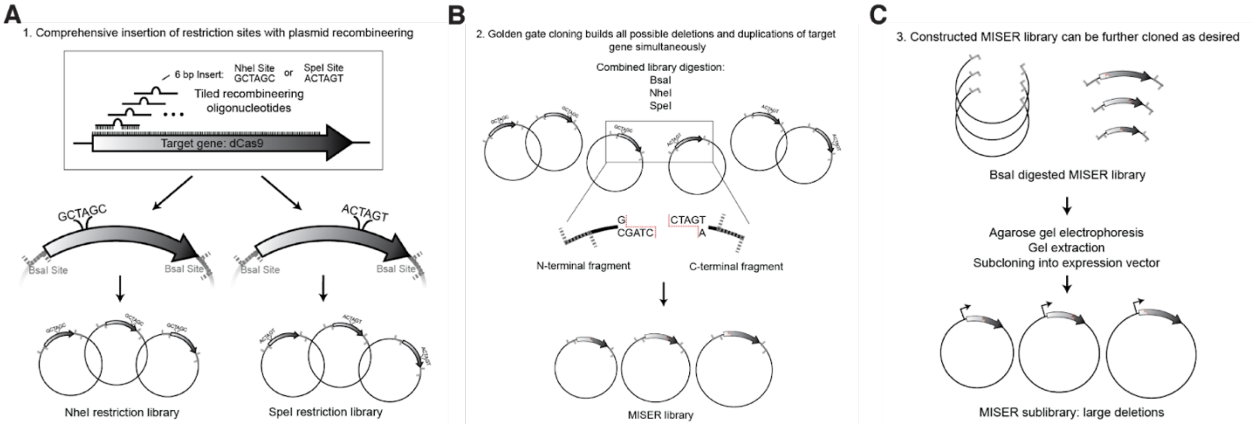

<u>Supplementary Figure 1:</u> Full cloning scheme for Minimization by Iterative Size-Exclusion and Recombination (MISER). The method can be considered in three parts. **A)** Plasmid recombineering generates two comprehensive libraries of restriction site insertions across the target gene. These restriction sites are both novel to the target plasmid and produce compatible sticky ends. Recombineering was performed similarly as in (Higgins 2017), where the target gene lacks a promoter and start codon to prevent growth biases during library construction and is flanked by BsaI sites for later Golden Gate cloning (here, plasmid pSAH060). Additionally, rather than mutagenic oligos, double stranded PCR product was used for recombineering, and another cloning step was introduced to remove unmodified plasmids. These modifications are described in Experimental Design. **B)** Modified golden gate cloning generates a library of ligated N- and C- terminal fragments of the target gene, comprehensively producing protein deletion variants as well as duplication variants. An equimolar mixture of the two plasmid libraries is mixed and fully digested to produce free N- and C- terminal fragments of the target gene. This fragment mixture is then re- ligated in the presence of NheI and SpeI. Successful ligation of an N- and C-terminal fragment from differing libraries produces one of two possible 6 base-pair scar sequences. These novel scar sequences are not recognized by either NheI or SpeI, thus trapping the desired chimeric product as a final ligated vector. Because N- and C-terminal fragments are ligated randomly, these chimeric products produce both protein deletions and protein duplications. Ideally the library is both large enough and minimally biased to produce a large fraction of possible variants. The product of this step can be considered a MISER library of plasmid pSAH060. **C)** A final cloning step moves the MISER library into a desired context – i.e. an expression plasmid, here pSAH063. Step C also allows for size-based exclusion of undesired protein variants by extraction from an agarose gel (Figure 1 and Supplementary Figure 2).

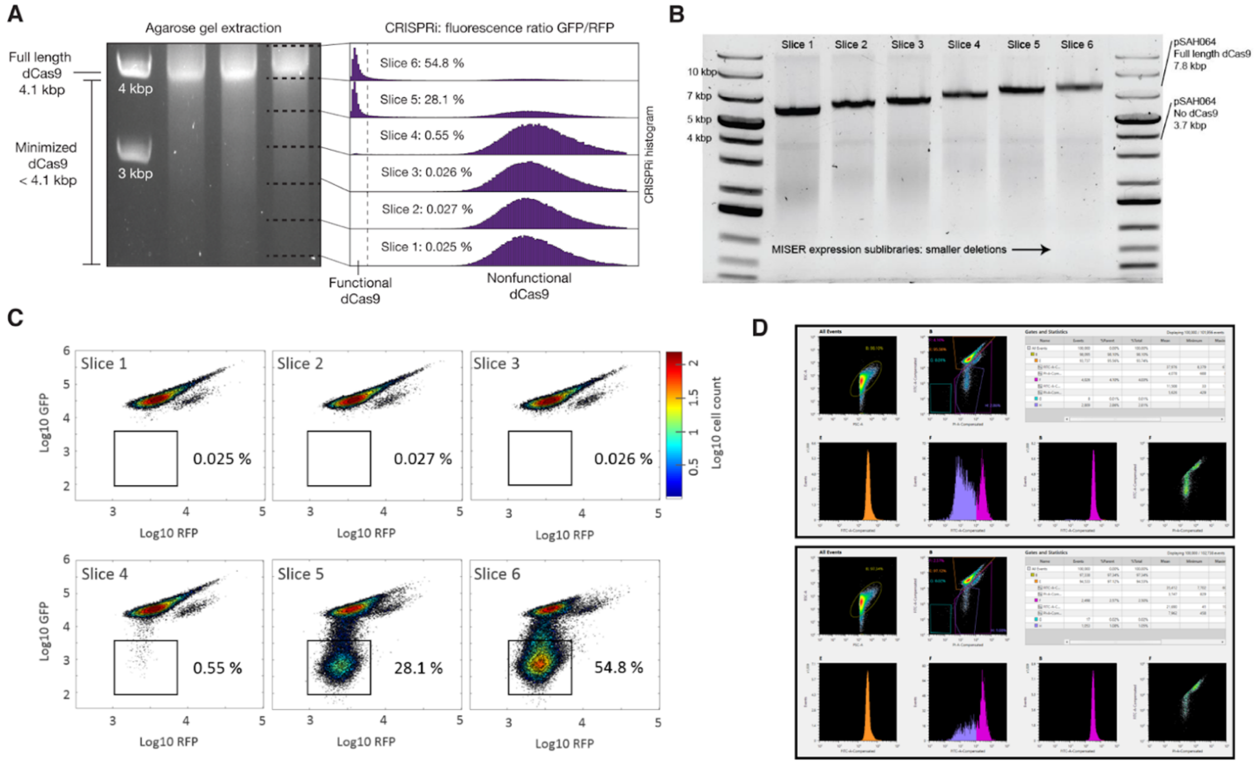

Supplementary Figure 2: **Size exclusion and flow cytometry identify the range of dCas9 deletion sizes exhibiting *in vivo* transcriptional repression. A)** To empirically determine the size range of functional deletions, an agarose gel of the dCas9 MISER deletion library was sliced into six sub-libraries, independently cloned into expression vectors (B), and assayed for CRISPRi GFP repression via flow cytometry (C). Sublibrary Slice 4 was the most stringent library with detectable repression, with functional variants becoming more frequent in slices composed of smaller deletions as expected. Agarose gel extraction of the six sub-libraries was performed once. **B)** The six gel slices in (A) were individually gel extracted and ligated into expression vector pSAH063, generating pSAH064 plasmids with dCas9 deletions. The resulting expression sub-libraries exhibit high precision in size ranges when assayed by agarose gel electrophoresis. Expression vector ligation with the six sub-libraries was performed once. **C)** Flow cytometry identifies Slice 4, 5, and 6 as expression sub-libraries containing functional dCas9 deletion variants. GFP repression CRISPRi was performed as described in Experimental Design. The region of phenotype defined as 'functional' is illustrated. The percent of functional hits is annotated. **D)** Screenshots from Sony Cell Sorter Software exemplifying the gating strategy, with upper panel showing full library sort and lower panel showing Slice 4. Gate H was used to sort cells containing repression-competent CE variants.

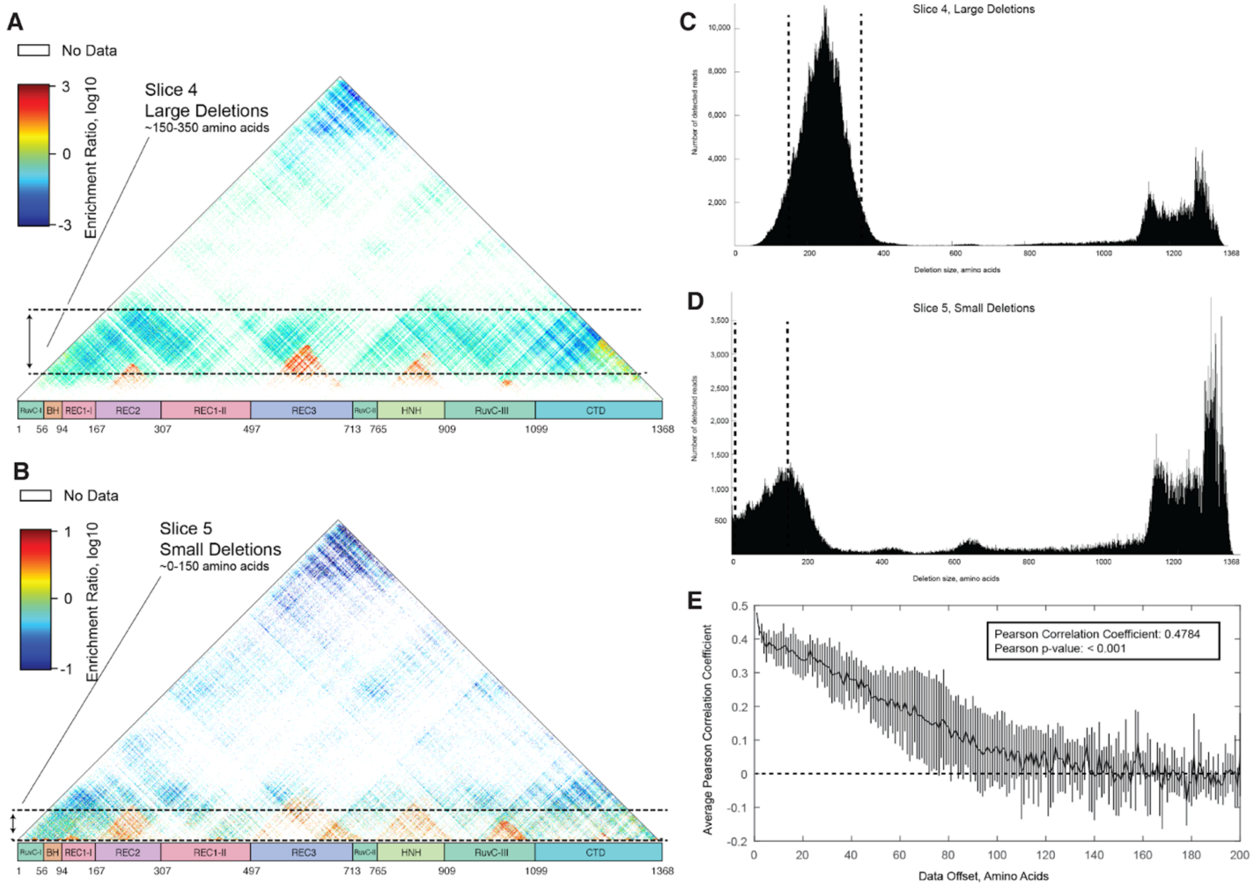

Supplementary Figure 3: Deep sequencing of the sublibraries of Slice 4 and Slice 5 reveal deletion regions throughout dCas9. A) Raw enrichment map of Slice 4 sub-library. Each pixel represents a single deletion variant, whose start and end points are the axis intercepts when moving down and to the left or right, respectively, as described in the main text. Domain boundaries are labeled by amino acid number. The pixel color also denotes the degree of enrichment or loss following flow cytometry screening for transcriptional repression in vivo. Detailed calculations are described in the supplementary methods. Deletions corresponding to sizes within the gel slice are indicated by dashed lines. **B)** Raw enrichment map of Slice 5 sub-library, as in (A). Note the differing range of enrichment ratios. **C)** Histogram of deletion sizes in the naïve Slice 4 library. The hypothetical edges of the gel slice are indicated by dashed lines. **D)** Histogram of deletion sizes in the naïve Slice 5 library. The edges of the gel slice are indicated by dashed lines. **E)** Slices 4 and 5 independently replicate the same large functional deletion regions. The raw enrichment maps of Slice 4 and Slice 5 contain many of the same variants, and a two-sided *t*-test of the Pearson correlation for these variants is highly significant ($p = 2.18e\text{-}290$). Furthermore, this correlation is progressively lost if the two enrichment maps are shifted relative to one another. The line plots the mean of four additional Pearson correlations where the data array has been offset – either up, down, left, or right – by the indicated number of amino acids. This analysis verifies that the two enrichment maps independently identify large-scale regions of dCas9 which can be deleted and validates the apparent visual correspondence between maps A and B. Data are presented as mean±SD. Source data are provided as a Source Data file.

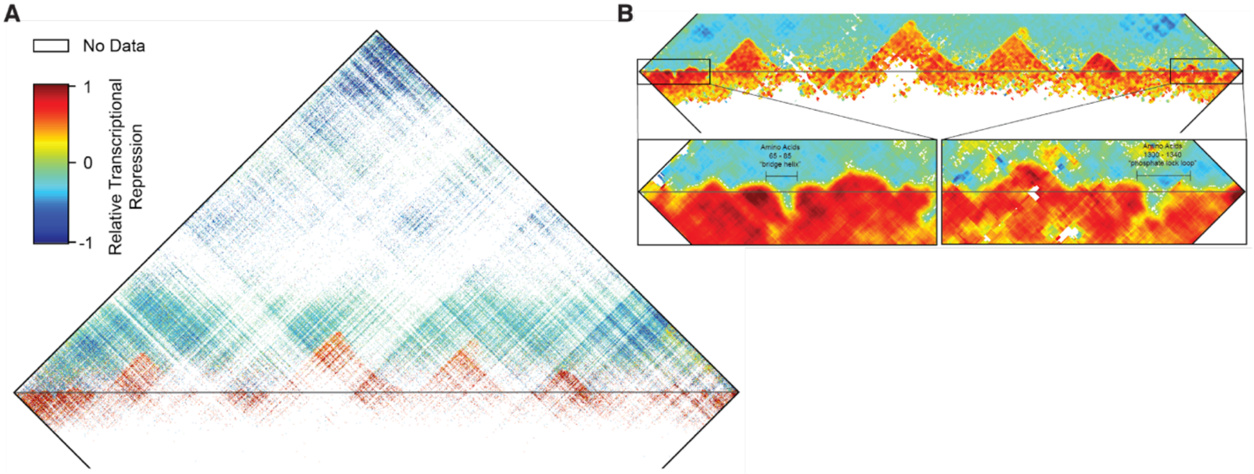

<u>Supplementary Figure 4:</u> Key elements of dCas9 secondary structure are revealed by the functional impact of small deletions and insertions. A) The enrichment map of Figure 1C is presented in its entirety, including small duplications of dCas9 sequence. The horizontal grey line corresponds to the boundary between deletions (top) and tandem duplicate insertions (bottom). Note that in all cases a two amino acid MISER scar is also present (either Ala-Ser or Thr-Ser) which is not included in display or numbering. **B)** The combined enrichment map in (A) was interpolated to highlight the boundaries between functional and non- functional deletions, which are not clearly visible in the raw data. Pixels were replaced by the mean enrichment value of neighboring deletions/duplications, plus itself, in a square window 10 amino acids wide. Windows with fewer than five values were left white. Insets: The N- and C- terminal regions were particularly well resolved by this method, and elements of interest are annotated. The 'bridge helix' and 'phosphate lock loop' are two examples of secondary structure which strongly disallow small insertions.

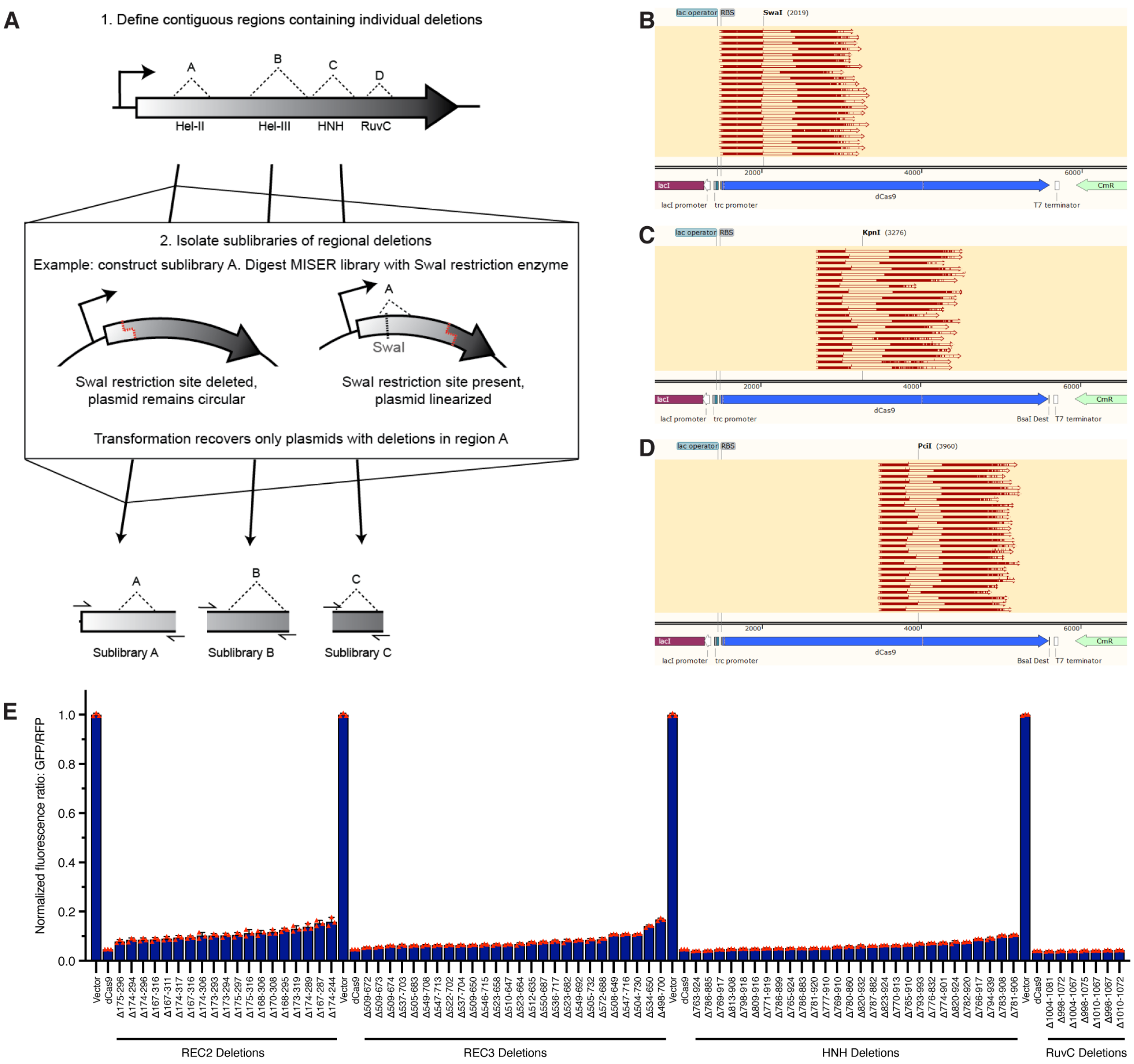

Supplementary Figure 5: MISER sublibraries composed of specific deletions can be generated by restriction digestion. A) Digesting a MISER library with a restriction enzyme that has exactly one site within the plasmid will linearize the majority of plasmids, while plasmids with the site deleted will remain circular. This reaction can then be transformed in order to recover a sublibrary containing deletions from a specific region. **B)** For example, the restriction enzyme SwaI was used to isolate deletions in the REC2 region. The enzyme recognition site is shown mapped to the sequence of pSAH064, the dCas9 expression plasmid, illustrating the overlap with various sequenced deletions. **C)** The restriction enzyme KpnI was used to isolate deletions in the REC3 region, as in B. **D)** The restriction enzyme PciI was used to isolate deletions in the HNH region, as in B. **E**) Sublibraries containing regional individual deletion variants were re- transformed, and colonies were picked and assayed for CRISPRi activity. A subset of the most active clones was Sanger sequenced to identify the precise deletion. RuvC deletions could not be isolated by the sublibrary approach, and instead were cloned manually by PCR. Data are plotted as mean±SD from biological triplicates. Source data are provided as a Source Data file.

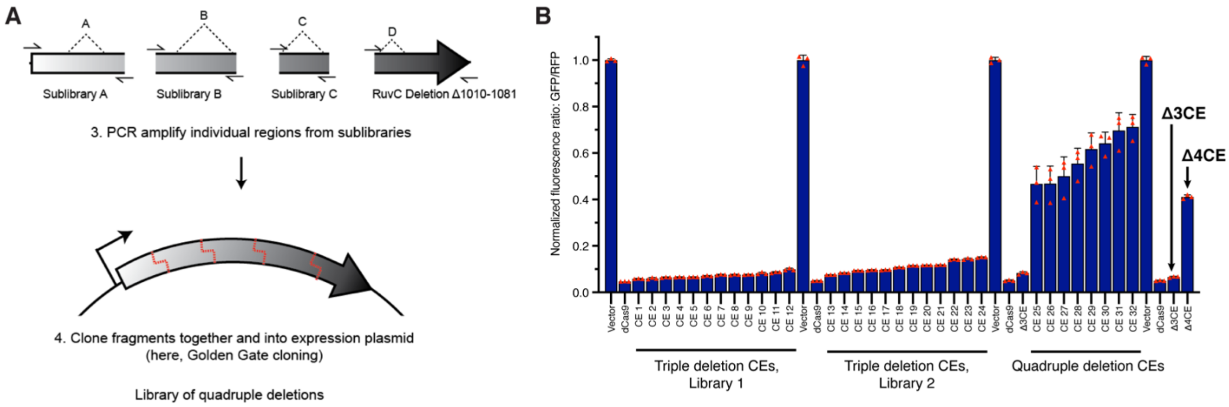

<u>Supplementary Figure 6:</u> Golden Gate Cloning builds libraries of CRISPR Effector (CE) variants with multiple deletions. A) One highly functional RuvC deletion variant from Region D was PCR amplified, along with Sublibraries A, B, and C. PCR primers added Golden Gate compatible sticky ends, enabling Golden Gate cloning of individual fragments to form a library of CE deletion variants, Library 1. **B)** Flow cytometry was performed to isolate the most functional CE variants from the "stacked" library described in (A). All highly functional CE variants from Library 1 were found to lack REC2 deletions (sequences of CE variants selected for display on this plot can be found in Table S3). To verify this result, a second version of Sublibrary A was created, using a different strategy to isolate REC2 deletions as follows: the full MISER library was digested with the restriction enzyme BlpI, which cuts at amino acids 227-228 (instead of SwaI), and the resulting DNA was used directly as template for the PCR reaction (BlpI cuts pSAH064 three times and thus cannot be directly re-transformed to isolate the sublibrary). Library 2 thus contains all four deletion variants as in Library 1, except the sublibrary of REC2 deletions was entirely remade. However, once again functional CE variants isolated by FACS lacked REC2 deletions. The most functional variant in Library 2, CE 13, was named Δ3CE. Finally, to directly assay the effects of a REC2 deletion, the REC2 region of Δ3CE was replaced with a library of deletions from Sublibrary A. These quadruple deletion CE variants all exhibited vastly reduced CRISPRi activity compared to Δ3CE alone. The most functional variant assayed was named Δ4CE. Data are plotted as mean±SD from biological triplicates. Source data are provided as a Source Data file.

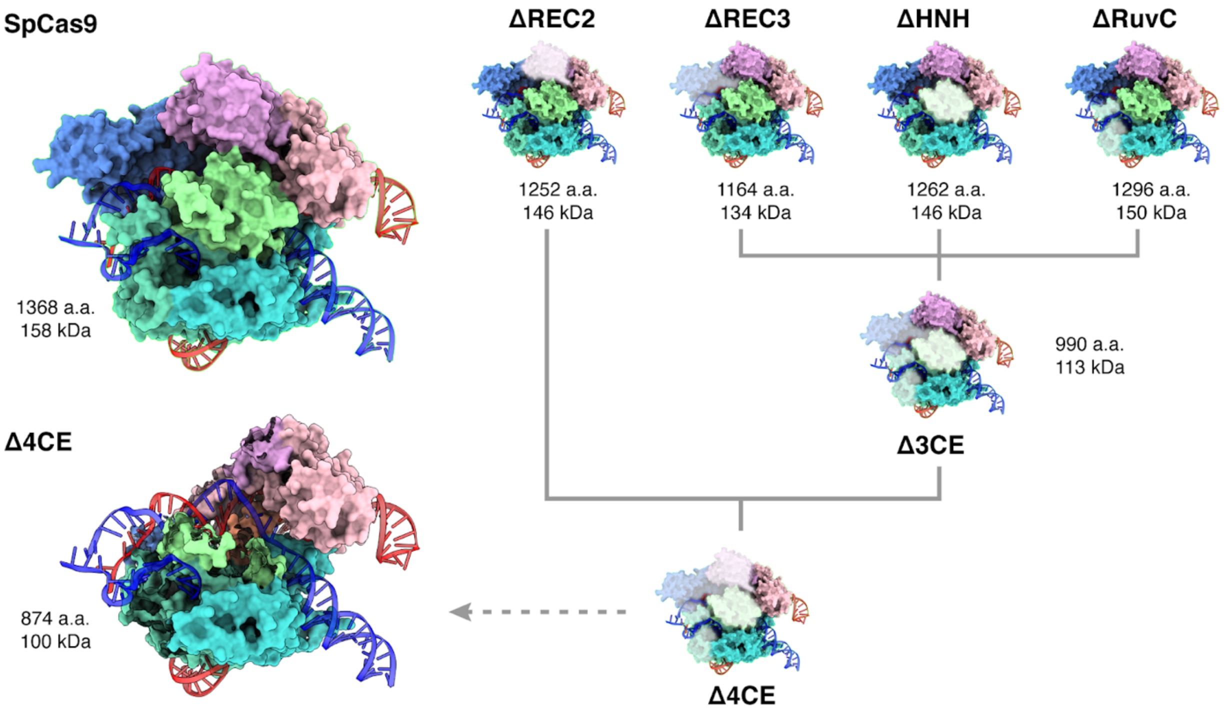

<u>Supplementary Figure 7:</u> 3D comparison of complete dCas9-sgRNA-dsDNA complex and modeled MISER constructs. Model of SpCas9 complexed with sgRNA and dsDNA (PDB 5Y36), and MISER domain deletions overlaid. Δ3CE contains the REC3, HNH, and RuvC deletions, and Δ4CE contains the additional REC2 deletion, as described in Fig. 2 and S5. The Δ4CE model is shown with the domains corresponding to MISER deletions hidden. Molecular weights are calculated by the ExPASy ProtParam tool (https://web.expasy.org/protparam/).

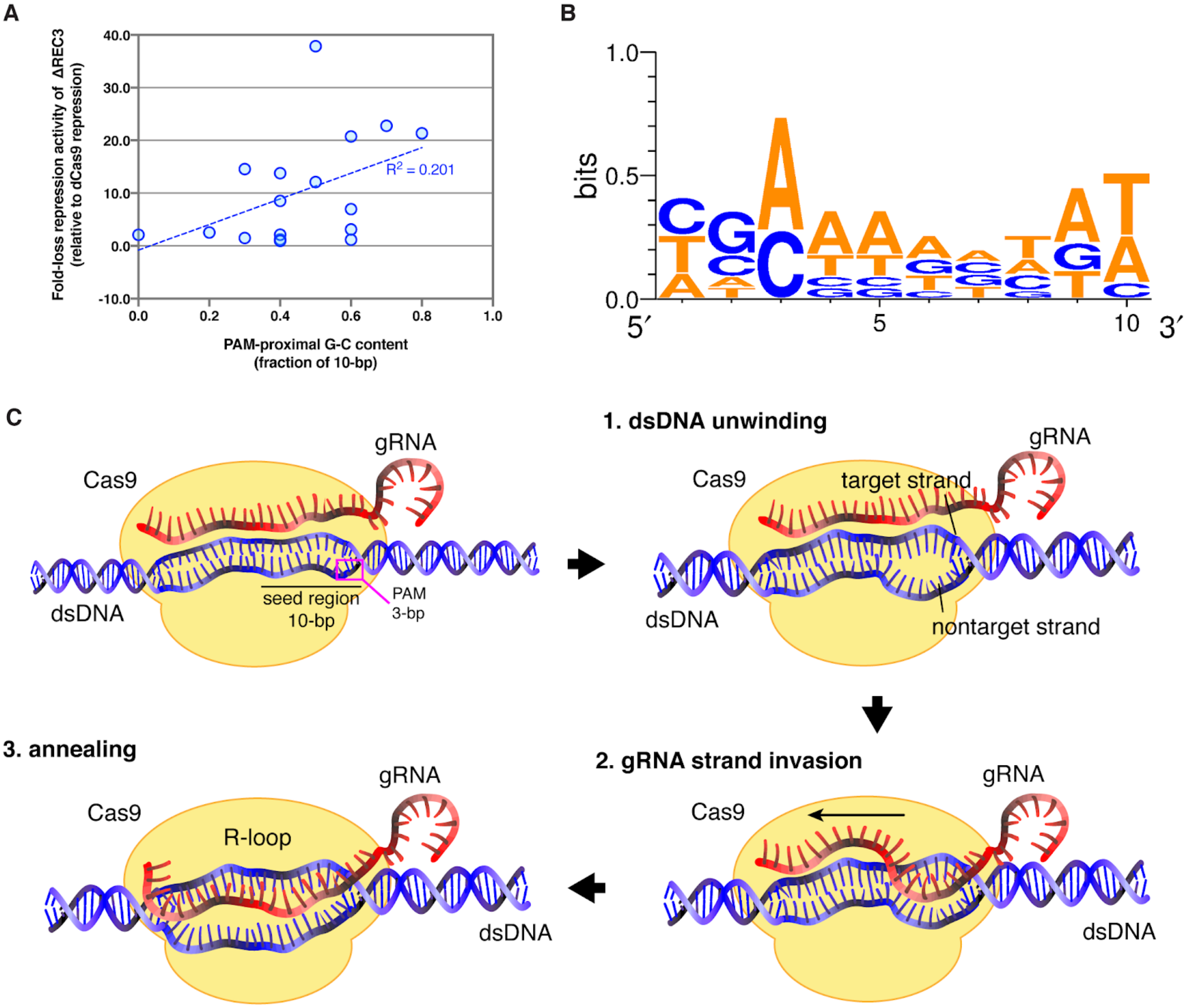

<u>Supplementary Figure 8:</u> Spacer sequence-dependent variability in repression activity of ΔREC3. A) Plot showing fold-change in repression by ΔREC3 for different targets versus fraction of G-C content in seed region. Correlation between G-C content and repression is low and does not fully explain the variability in repression seen by the ΔREC3 construct across different target sequences. Oligos used to generate plot are shown in Supplementary Table 4. **B)** WebLogo showing spacer sequence variability for guides that exhibit at least a three-fold loss in repression by ΔREC3 compared to dCas9. Oligos used to generate WebLogo are indicated in Supplementary Table 4. **C)** Schematic showing the process of gRNA invasion into the dsDNA target leading to R-loop formation by Cas9. In Step 1, unwinding of the dsDNA double-helix is initiated at 1-2 bases adjacent to the PAM in the seed region, creating a destabilized region where the gRNA can invade, in Step 2. Hybridization of the gRNA to the target strand occurs in the seed region and proceeds in the PAM-distal direction (3’→5’), until the entire spacer sequence (~20bp) is annealed to the target strand, generating an RNA-DNA duplex called an R-loop (Step 3). RNA-DNA hybrid is shown as a 2-D representation for clarity instead of a helix.

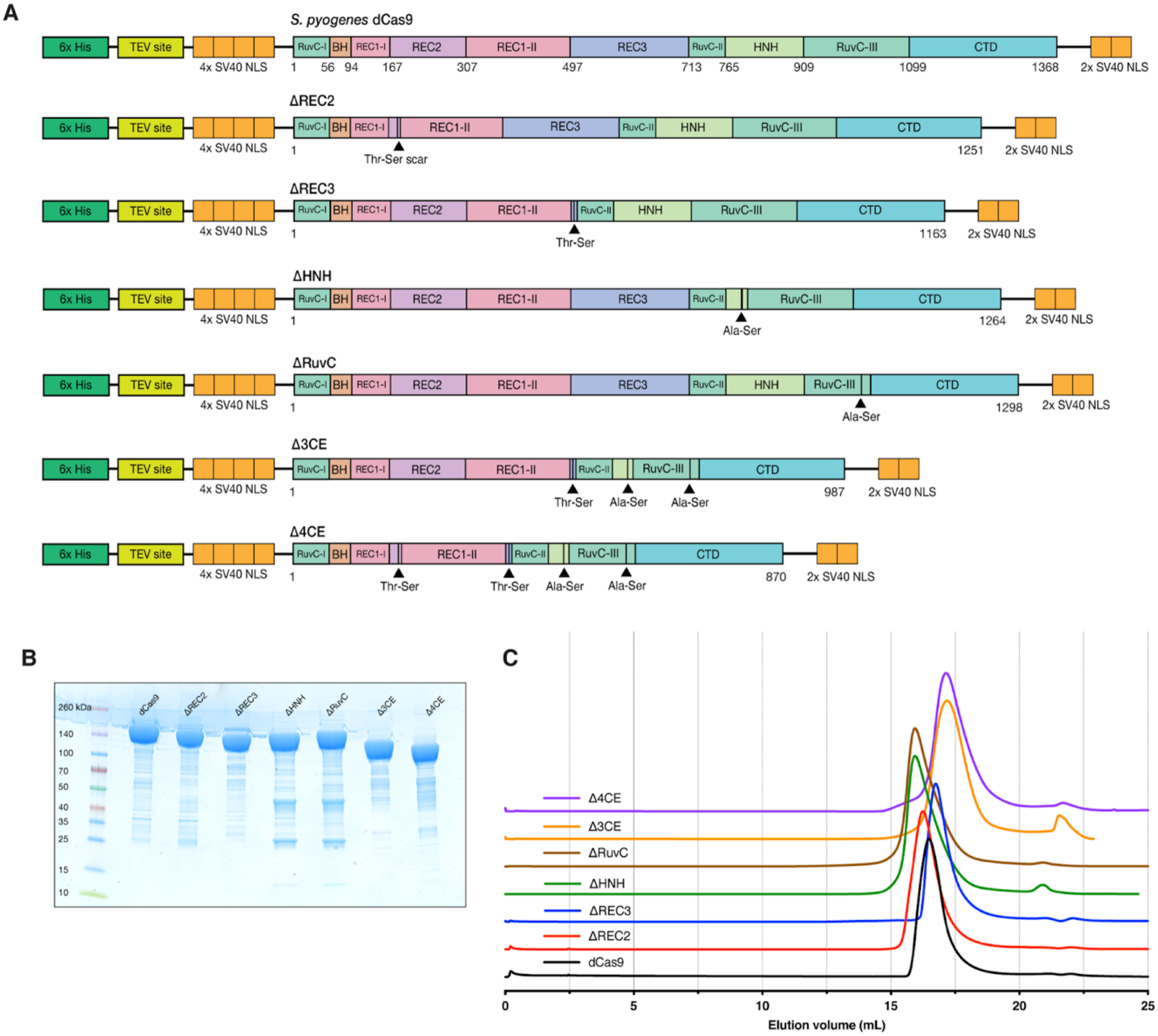

Supplementary Figure 9: Expression constructs and protein purification of MISER constructs. A) Expression constructs for dCas9 containing MISER deletions and accompanying scars. All constructs were expressed using an IPTG-induced T7 promoter, and contain a N-terminal 6x His-tag, a TEV protease site, 4x SV40 NLS, and 2x SV40 NLS on the C-terminus. **B)** Representative SDS-PAGE of purified MISER constructs from at least two purifications. **C)** Size-exclusion chromatogram showing elution of all MISER constructs on a GE Superose 6 Increase column. Source data are provided as a Source Data file.

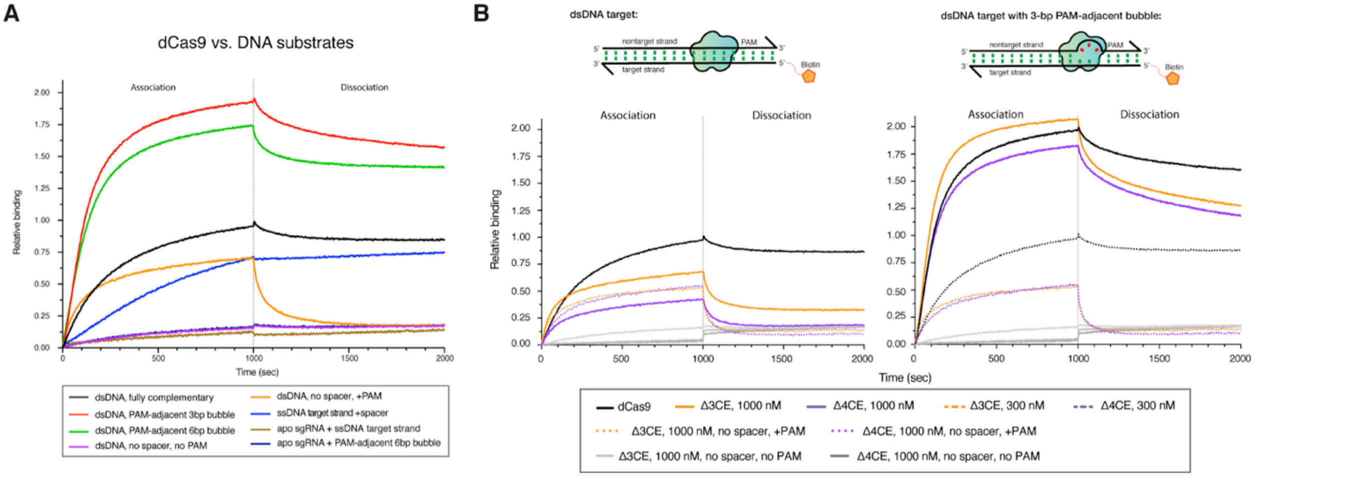

<u>Supplementary Figure 10:</u> Bio-layer interferometry (BLI) controls. A) BLI experiments were performed by incubating immobilized dCas9 with dsDNA containing a target spacer but no PAM (orange trace). Transient PAM interactions have a significant contribution to the k_{on} of association. The signal is lost immediately in the dissociation step, which suggests that the interaction is nonspecific. Conversely, incubation with a dsDNA containing no spacer and no PAM shows no signal (purple). **B)** BLI traces of Δ3CE and Δ4CE binding to dsDNA show that the relative binding is minimal at 300 nM, even with a 3-bp bubble in the seed region of the target (orange and purple). Subsequently a concentration of 1000 nM was used for these constructs. Dotted lines represent Δ3CE and Δ4CE RNPs interacting with a target without complementary spacers but containing NGG PAMs. Light grey and dark grey traces represent Δ3CE and Δ4CE RNPs, respectively, against dsDNA without a spacer or PAM. All data shown are normalized to the maximum signal of dCas9 vs. fully complementary dsDNA target (black). Source data are provided as a Source Data file.

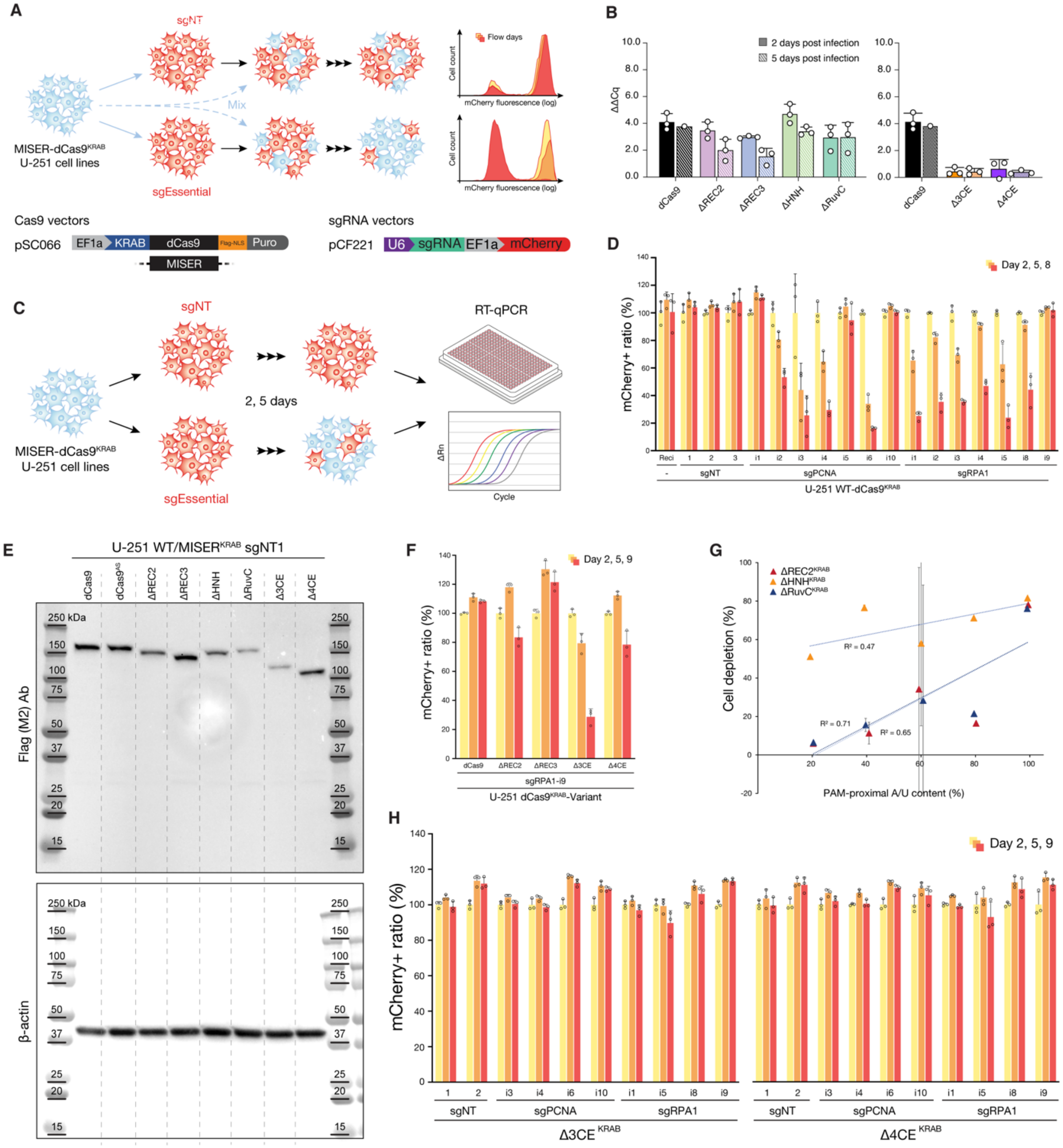

<u>Supplementary Figure 11:</u> Schematic of CRISPR interference (CRISPRi) based survival assay. A) Schematic of CRISPR interference (CRISPRi) based competitive proliferation assay. U-251 glioblastoma cells are stably transduced with lentiviral vectors (pSC066) expressing MISER-dCas9 or WT-dCas9 KRAB fusion proteins, followed by selection on puromycin. The various cell lines are then transduced with a secondary lentiviral vector (pCF221) expressing mCherry fluorescence protein and either sgRNAs targeting essential genes (sgEssential) or non-targeting sgRNAs (sgNT) as controls. After mixing with the respective parental population, the percentage of mCherry-positive cells is monitored by flow cytometry over several days. **B)** PCNA $\Delta\Delta C_q$ values from RT-qPCR at 2 (solid) and 5 (hatched) days post infection, calculated by subtracting target samples from sgNT samples. Data presented as mean±SD (for triplicates where shown). **C)** U-251 glioblastoma cells are stably transduced with lentiviral vectors (pSC066) expressing MISER-dCas9 or WT-dCas9 KRAB fusion proteins, followed by selection on puromycin. The various cell lines are then transduced with a secondary lentiviral vector (pCF221) expressing mCherry fluorescence protein and either sgRNAs targeting essential genes (sgPCNA) or non-targeting sgRNAs (sgNT) as controls. Cells

are grown and harvested 2- and 5-days post-infection for RNA extraction, followed by RT-qPCR to quantitate transcription of targeted essential genes under MISER-KRAB repression. **D)** U-251 cells stably expressing a wild-type dCas9 KRAB fusion protein (WT-dCas9-KRAB) were transduced with lentiviral vectors expressing the indicated sgRNAs. At Day-2 post-transduction, cells were mixed with the parental population; mCherry fluorescence was monitored over time. sgNT, non-targeting control sgRNAs. sgPCNA and sgRPA1, sgRNAs targeting essential genes. Reci, recipient vector for sgRNA cloning. Data represent the mean and standard deviation of triplicates (n=3). **E)** Immunoblotting for Flag-tagged MISER-dCas9 or WT-dCas9 KRAB fusion proteins stably expressed in U-251 cells co-expressing a non-targeting guide (sgNT1). The indicated MISER deletions result in reduction of protein size. $dCas9^{AS}$ represents an alternative out-of-frame start-codon derived from the native sequence of the KRAB domain. Beta-actin (ACTB) was used as loading control. Protein ladders indicate reference molecular weight markers in kDa. Experiment was carried out once. **F)** Competitive proliferation assay as in (D). Note, the indicated sgRNA (sgRPA1-i9) shows stronger depletion with some of the MISER variants when compared to the WT-dCas9 KRAB fusion. Significance in increased cell depletion was assessed by comparing samples to the wild-type control using unpaired, two-tailed t-tests (alpha = 0.01). Data represent the mean and standard deviation of triplicates (n=3). **G)** Correlation between PAM-proximal A/U content of sgRNAs (5 most proximal bases) and cell depletion efficiency at day 9 of the competitive proliferation assay for the indicated MISER-dCas9 KRAB fusion variants. The scatter plot represents data from sgPCNA-i3/i4/i6 and sgRPA1-i1/i5/i8/i9. Dotted lines indicate linear regressions (ΔREC2 $R^2 = 0.65$, ΔHNH $R^2 = 0.47$, ΔRuvC $R^2 = 0.71$). **H)** Competitive proliferation assay as in Fig 1E, with stacked-deletion constructs Δ3CE and Δ4CE. Data represent the mean and standard deviation of triplicates (n=3). Significance in cell depletion was assessed by comparing samples to their respective Day-2 controls using unpaired, two-tailed t-tests (alpha = 0.01). Source data are provided as a Source Data file.

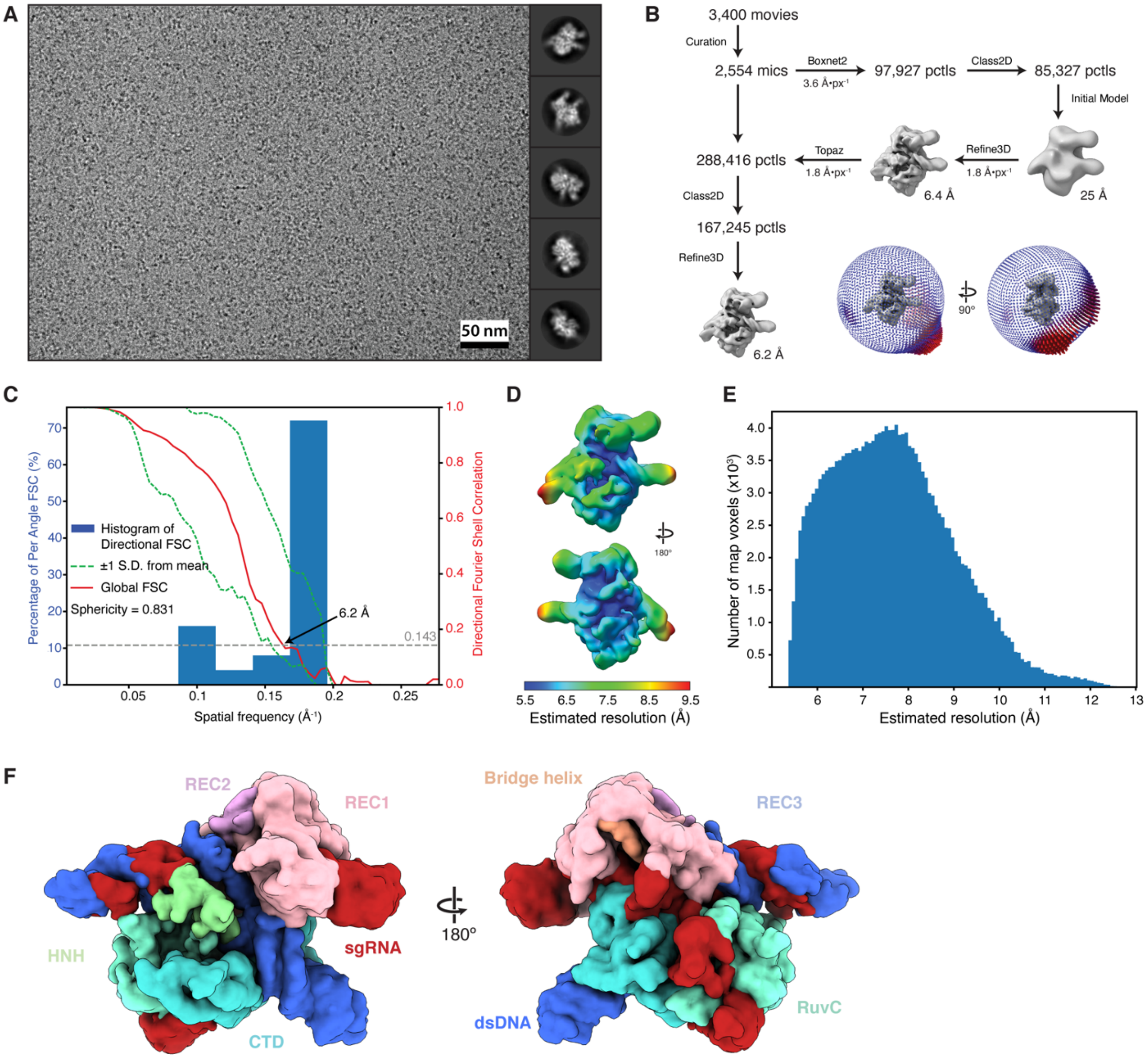

<u>Supplementary Figure 12:</u> Single-particle cryo-EM of the Δ4Cas9 ternary complex. A) Exemplar micrograph at approximately 3 microns defocus with scale indicated and representative reference-free 2D class averages from the Topaz-picked particle set. A total of 3400 micrographs were collected, of which 2,554 were used (panel B). Diameter of 2D mask is 150 Å in all averages. All cryo-EM data were collected from a single grid. No statistical methods were used to predetermine sample size. **B)** Single-particle reconstruction workflow as described in methods and orientation distribution of the final reconstruction inset. (C) Directional FSC for final reconstruction. **D) and E)** Local resolution estimates calculated in RELION shown by coloration on the map and as a histogram, respectively. Source data are provided as a Source Data file. **F)** Density map of Δ4CE with putative domains segmented and colored according to their relative position within a 20 Å radius when overlaid on WT SpCas9 (PDB 5Y36).

	**SpeI Insertion**	**NheI Insertion**
**Recombineering Oligo: Insertion Site 1**	AACACGTCCGTCCTAGAACTcgtctcatacgcaaAccgcctctccccgcgcgttggcggtctcaatctATGactagtgataagaaatactcaataggcttagctatcggcacaaatagcgtcgggagacgGCAAGCGGTACACTCAGATCAGTGTTGAGCGTAACCAAGT	AACACGTCCGTCCTAGAACTcgtctcatacgcaaAccgcctctccccgcgcgttggcggtctcaatctATGgctagcgataagaaatactcaataggcttagctatcggcacaaatagcgtcgggagacgGCAAGCGGTACACTCAGATCAGTGTTGAGCGTAACCAAGT

Supplementary Table 1: **Example Oligo Library Synthesis (OLS) oligonucleotides used in this study.** The full list of ordered oligonucleotides is available as 'Supplementary Data 1 - Recombineering Oligonucleotides'. All oligonucleotides were ordered from Agilent Technologies, Inc. Oligos were designed to incorporate 45 and 47 bp of homology upstream or downstream of the insertion site, respectively (lowercase). Six bp were inserted between dCas9 codons, beginning after the target codon. The above example targets the start codon, 'ATG' (bold uppercase). These six bp consisted of recognition sequences for either the restriction enzyme SpeI or NheI (underlined). Flanking primer sequences allowed the amplification of the entire OLS library (italics) using oligonucleotides SAH_284 and SAH_285 (Table S6). Specific libraries of SpeI recombineering oligonucleotides or NheI recombineering oligonucleotides were amplified using forward primer SAH_284 and either SAH_286 or SAH_287 reverse primers, respectively. After amplification, these dsDNA products can be 'matured' by cleavage with the restriction enzyme BsmbI (bold lowercase), which cleaves internally of its recognition site, thus removing all non-homologous priming sequence from the recombineering template.

Deletion	Δ3CE v1	Δ3CE v2	Δ3CE v3	Δ3CE v4	Δ3CE v5	Δ3CE v6	Δ3CE v17	Δ3CE v21	Δ3CE v22	Δ3CE	Δ4CE
**REC2**	-	-	-	-	-	-	-	-	-	-	[180-297]
**REC3**	[511-716]	[498-699]	[500-688]	[497-700]	[501-664]	[512-721]	[509-650]	[508-649]	[508-646]	[503-708]	[503-708]
**HNH**	[813-909]	[813-908]	[811-898]	[786-882]	[804-893]	[809-916]	[776-923]	[768-900]	[786-923]	[792-897]	[792-897]
**RuvC**	[1010-1081]	[1010-1081]	[1010-1081]	[1010-1081]	[1010-1081]	[1010-1081]	[1010-1081]	[1010-1081]	[1010-1081]	[1010-1081]	[1010-1081]

Supplementary Table 2: Deletions present in selected MISER variants. Indicated numbers represent the first and last amino acid deleted from the protein.

	**Total Reads**	**Deletions Sequenced**	**Unique Deletions**	**Enriched Unique Deletions**	**De-enriched Unique Deletions**
**Slice 4 Naïve**	132,274,232	1,923,543	192,447		
**Slice 4 Sorted**	140,589,968	1,960,138	25,948	19,618	6,330
**Slice 5 Naïve**	37,873,068	590,859	111,438		
**Slice 5 Sorted**	35,016,326	290,947	51,462	31,794	19,668
**Total**	345,753,594	4,765,487	381,295	51,412	25,998

Supplementary Table 3: Statistics for deep sequencing of MISER libraries Slice 4 and Slice 5.

Gene	Distance from RBS (bp)	PAM-proximal 10bp sequence (5'-3')	PAM-proximal G-C fraction	Fold loss	Std. dev.
**GFP**	**38**	**AACAAGAATT-NGG**	**0.2**	**2.54**	**0.23**
RFP	124	TTAGCGGTCT-NGG	0.5	37.84	3.78
**GFP**	**130**	**ATAAATTTAA-NGG**	**0.0**	**2.11**	**0.01**
**GFP**	**174**	**TGACAAGTGT-NGG**	**0.4**	**1.23**	**0.02**
**GFP**	**196**	**TGAACACCAT-NGG**	**0.4**	**2.14**	**0.10**
**GFP**	**225**	**TCATGTGATC-NGG**	**0.4**	**0.96**	**0.05**
GFP	262	CCTTCGGGCA-NGG	0.7	22.77	0.73
**GFP**	**316**	**CGCGTCTTGT-NGG**	**0.6**	**1.18**	**0.06**
**GFP**	**355**	**CGATTAACAA-NGG**	**0.3**	**1.50**	**0.06**
RFP	111	TACCTTCGTA-NGG	0.4	8.54	0.50
RFP	130	TTCAGTTTAG-NGG	0.3	14.56	0.77
**RFP**	**165**	**CCCAAGCGAA-NGG**	**0.6**	**3.13**	**0.06**
RFP	182	CTGCGGGGAC-NGG	0.8	21.35	0.71
RFP	197	GGAACCGTAC-NGG	0.6	6.98	0.23
RFP	208	ACGTAAGCTT-NGG	0.4	13.79	2.92
RFP	239	CAGGTAGTCC-NGG	0.6	20.74	4.25
RFP	248	GGACAGTTTC-NGG	0.5	12.10	0.60

Supplementary Table 4: gRNA target loci and G-C content dependence of ΔREC3 repression. Spacer sequences highlighted in blue were used to generate the WebLogo in Supplementary Figure 9A.

**EMDB-22518**	
**Data Collection**	
Microscope	Talos Arctica
Magnification	45,000
Voltage (kV)	200
Detector	K3
Electron exposure (e-/Å^2)	60
Defocus range (µm)	1.5 to 3.8
Pixel size (Å)	0.45[a]
**Reconstruction**	
Symmetry imposed	C1
Box size (pixels/Å)	128/230
Initial particle images (no.)	288,416[b]
Final particle images (no.)	167,245
Map resolution (Å)	6.2
FSC threshold	0.143
Sharpening factor (Å^2)	-395
Map resolution range (Å)	5.5-9.5
Sphericity	0.831
**Modeling**	
Method	Rigid-body
Initial Model	5Y36
CC	0.73

[a]Super-resolution
[b]from picking with Topaz

Supplementary Table 5: Cryo-EM data collection & reconstruction statistics.

Oligo ID	Purpose	Sequence (5'-3')
SAH_284	Recombineering amplification: universal forward	AACACGTCCGTCCTAGAACT
SAH_285	Recombineering amplification: universal reverse	ACTTGGTTACGCTCAACACT
SAH_286	Recombineering amplification: SpeI-specific reverse	GATCTGAGTGTACCGCTTGC
SAH_287	Recombineering amplification: NheI-specific reverse	GATCGCCTAGACAACTCCTG
sgRNA-B9	sgRNA for Cas9 RNP, used in BLI and cryo-EM	AGUCGGUGUCGACCCGGACCCAAAAUCUCGAUC UUUAUCGUUCAAUUUUAUUCCGAUCAGGCAAUAG UUGAACUUUUUCACCGUGGCUCAGCCACGAAAA
oAS081	5'-biotinylated ssDNA target for BLI, sgRNA-B9	GCTCAATTTTGACAGCCCACCAGGCCCAGCTGTG GCTGATGGCATCCTTCCACTC
oAS003a	non-target ssDNA for BLI (complementary to oAS081)	GAGTGGAAGGATGCCATCAGCCACAGCTGGGCCT GGTGGGCTGTCAAAATTGAGC
oAS114	5'-biotinylated ssDNA non-target for BLI (no spacer, no PAM)	GTGTGCACACATGCAATAACATTGTGCACATGATA CATTGCAATGACAATTAACC
oAS036	non-target ssDNA for BLI (complementary to oAS081, 3-bp PAM-proximal bubble)	GAGTGGAAGGATGCCATCAGCCACAGCTGGGCC GATTGGGCTGTCAAAATTGAGC
oAS116	unlabeled ssDNA target for BLI, sgRNA-B9. Used for cryo-EM RNP complex	GCTCAATTTTGACAGCCCACCAGGCCCAGCTGTG GCTGATGGCATCCTTCCACTC
sgNT-1	Non-targeting gRNA for mammalian CRISPRi	GGCCAAACGTGCCCTGACGG
sgNT-2	Non-targeting gRNA for mammalian CRISPRi	GCGATGGGGGGGTGGGTAGC
sgPCNA-i1	*PCNA* targeting gRNA for mammalian CRISPRi	GGGGCGAACGTCGCGACGAC
sgPCNA-i2	*PCNA* targeting gRNA for mammalian CRISPRi	GGCGTGGTGACGTCGCAACG
sgPCNA-i3	*PCNA* targeting gRNA for mammalian CRISPRi	GCGCTCCCGCCAAGCACCGG
sgPCNA-i4	*PCNA* targeting gRNA for mammalian CRISPRi	GAAGCGCTCCCGCCAAGCAC
sgPCNA-i5	*PCNA* targeting gRNA for mammalian CRISPRi	GCCCGGCCCGCCTGCACCTC
sgPCNA-i6	*PCNA* targeting gRNA for mammalian CRISPRi	GCGGACGCGGCGGCATTAAA
sgPCNA-i10	*PCNA* targeting gRNA for mammalian CRISPRi	GGCCATCCGCGCCTTCTCAT
sgRPA1-i1	*RPA* targeting gRNA for mammalian CRISPRi	GGGAAGCTGGAGCTGTTGCG
sgRPA1-i2	*RPA* targeting gRNA for mammalian CRISPRi	GGCGACGGGGGATGAACGCG
sgRPA1-i3	*RPA* targeting gRNA for mammalian CRISPRi	GTGCGCAGCGCGCGGGACCC

sgRPA1-i4	*RPA* targeting gRNA for mammalian CRISPRi	GTGAGCCGCGCGCACGTCGG
sgRPA1-i5	*RPA* targeting gRNA for mammalian CRISPRi	GGCGGTGCGCGCAACTTCTC
sgRPA1-i8	*RPA* targeting gRNA for mammalian CRISPRi	GCGAGCCTCGCGGAGTAGAG
sgRPA1-i9	*RPA* targeting gRNA for mammalian CRISPRi	GCCGCGCGCTGCGCAGTTAT
oAS085	Forward primer for *RPA1* cDNA reverse transcription, set 1	GCAGTTGGAGTGAAGATTGG
oAS086	Reverse primer for RPA1 cDNA RT, set 1	CACTTGGACTGGTAAGGAGT
oAS087	Forward primer for *RPA1* cDNA RT, set 2	CCGAGCTACAGCTTTCAATG
oAS088	Reverse primer for RPA1 cDNA RT, set 2	GCAGATCCCGATGATGTCTA
oAS089	Forward primer for *PCNA* cDNA RT, set 1	ACTCAAGGACCTCATCAACG
oAS091	Reverse primer for PCNA cDNA RT, set 1	TGAACCTCACCAGTATGTCC
oAS090	Forward primer for *PCNA* cDNA RT, set 2	CGTTATCTTCGGCCCTTAGT
oAS092	Reverse primer for PCNA cDNA RT, set 2	CGTGCAAATTCACCAGAAGG
oAS117	Forward primer for *GAPDH* RT	TCAAGGCTGAGAACGGGAAG
oAS118	Reverse primer for *GAPDH* cDNA RT	TGGACTCCACGACGTACTCA
oAS034	Forward primer for cloning dCas9 and MISER constructs into expression vector	GGTATCAACTTTTCGTTTCTT
oAS035	Reverse primer for cloning dCas9 and MISER constructs into expression vector	CAAAGCCCGAAAGGAAG

Supplementary Table 6: **Oligonucleotides used in this study.**

REFERENCES

1. Oakes, B. L. *et al.* CRISPR-Cas9 Circular Permutants as Programmable Scaffolds for Genome Modification. *Cell* **176,** 254–267.e16 (2019).
2. Zivanov, J., Nakane, T. & Scheres, S. H. W. Estimation of high-order aberrations and anisotropic magnification from cryo-EM data sets in RELION-3.1. *IUCrJ* **7,** 253–267 (2020).
3. Rohou, A. & Grigorieff, N. CTFFIND4: Fast and accurate defocus estimation from electron micrographs. *J. Struct. Biol.* **192,** 216–221 (2015).
4. Tegunov, D. & Cramer, P. Real-time cryo-electron microscopy data preprocessing with Warp. *Nat. Methods* **16,** 1146–1152 (2019).
5. Chen, S. *et al.* High-resolution noise substitution to measure overfitting and validate resolution in 3D structure determination by single particle electron cryomicroscopy. *Ultramicroscopy* **135,** 24–35 (2013).
6. Bepler, T., Noble, A. J. & Berger, B. Topaz-Denoise: general deep denoising models for cryoEM. *BioRxiv* (2019). doi:10.1101/838920
7. Tan, Y. Z. *et al.* Addressing preferred specimen orientation in single-particle cryo-EM through tilting. *Nat. Methods* **14,** 793–796 (2017).
8. Huai, C. *et al.* Structural insights into DNA cleavage activation of CRISPR-Cas9 system. *Nat. Commun.* **8,** 1375 (2017).
9. Goddard, T. D. *et al.* UCSF ChimeraX: Meeting modern challenges in visualization and analysis. *Protein Sci.* **27,** 14–25 (2018).
10. Ramlaul, K., Palmer, C. M. & Aylett, C. H. S. A local agreement filtering algorithm for transmission EM reconstructions. *J. Struct. Biol.* **205,** 30–40 (2019).
