# Supplementary material for: Comprehensive deletion landscape of CRISPR-Cas9 identifies minimal RNA-guided DNA-binding modules
Source: Nat Commun. 2021 Sep 27;12:5664. doi: 10.1038/s41467-021-25992-8 (PMC8476515; doi:10.1038/s41467-021-25992-8)
Supplement: Supplementary file 2 — Reporting Summary [file 41467_2021_25992_MOESM2_ESM.pdf]

nature research

Corresponding author(s): David F. Savage

Last updated by author(s): Aug 19, 2021

Reporting Summary

Nature Research wishes to improve the reproducibility of the work that we publish. This form provides structure for consistency and transparency in reporting. For further information on Nature Research policies, see our Editorial Policies and the Editorial Policy Checklist.

Statistics

For all statistical analyses, confirm that the following items are present in the figure legend, table legend, main text, or Methods section.

n/a	Confirmed	
☐	☒	The exact sample size (*n*) for each experimental group/condition, given as a discrete number and unit of measurement
☐	☒	A statement on whether measurements were taken from distinct samples or whether the same sample was measured repeatedly
☐	☒	The statistical test(s) used AND whether they are one- or two-sided<br>*Only common tests should be described solely by name; describe more complex techniques in the Methods section.*
☐	☒	A description of all covariates tested
☐	☒	A description of any assumptions or corrections, such as tests of normality and adjustment for multiple comparisons
☐	☒	A full description of the statistical parameters including central tendency (e.g. means) or other basic estimates (e.g. regression coefficient) AND variation (e.g. standard deviation) or associated estimates of uncertainty (e.g. confidence intervals)
☐	☒	For null hypothesis testing, the test statistic (e.g. *F*, *t*, *r*) with confidence intervals, effect sizes, degrees of freedom and *P* value noted<br>*Give P values as exact values whenever suitable.*
☒	☐	For Bayesian analysis, information on the choice of priors and Markov chain Monte Carlo settings
☒	☐	For hierarchical and complex designs, identification of the appropriate level for tests and full reporting of outcomes
☐	☒	Estimates of effect sizes (e.g. Cohen's *d*, Pearson's *r*), indicating how they were calculated

Our web collection on statistics for biologists contains articles on many of the points above.

Software and code

Policy information about availability of computer code

Data collection	Sony Cell Sorter Software v-2.1.5, Attune NxT Flow Cytometer Software v2.5, Octet Data Acquisition and Analysis HT v-10.0, SerialEM-v3.7
Data analysis	MATLAB R2019a, Expasy ProtParam tool (updated 2005), GraphPad Prism v-9.1.1, RELION-v3.1b, Topaz-v0.2.3, ChimeraX-v1.1, CCP-EM c-1.4.1. Sequence mapping was performed with custom code that has been made available on GitHub (https://github.com/savagelab).

For manuscripts utilizing custom algorithms or software that are central to the research but not yet described in published literature, software must be made available to editors and reviewers. We strongly encourage code deposition in a community repository (e.g. GitHub). See the Nature Research guidelines for submitting code & software for further information.

Data

Policy information about availability of data

All manuscripts must include a data availability statement. This statement should provide the following information, where applicable:

- Accession codes, unique identifiers, or web links for publicly available datasets
- A list of figures that have associated raw data
- A description of any restrictions on data availability

Naïve and sorted sequencing reads for Slices 4 and 5 can be accessed from NCBI GenBank; accession code PRJNA746606 [https://www.ncbi.nlm.nih.gov/bioproject/PRJNA746606]. Wild-type SpCas9 cryo-EM data was downloaded from the Electron Microscopy Data Bank (EMDB); accession code 8236 [https://www.ebi.ac.uk/pdbe/entry/emdb/EMD-8236]. The 3D model for the structure was obtained from the Protein Data Bank (PDB); entry 5Y36 [http://dx.doi.org/10.2210/pdb5y36/pdb]46. All sequencing data that support the findings of this study are available from the authors upon reasonable request. Cryo-EM data for the Δ4CE construct are available at EMDB; accession code 22518. All other relevant data are available from the corresponding author on request.

Field-specific reporting

Please select the one below that is the best fit for your research. If you are not sure, read the appropriate sections before making your selection.

☒ Life sciences ☐ Behavioural & social sciences ☐ Ecological, evolutionary & environmental sciences

For a reference copy of the document with all sections, see nature.com/documents/nr-reporting-summary-flat.pdf

Life sciences study design

All studies must disclose on these points even when the disclosure is negative.

Sample size	For libraries of protein variants, all cloning steps were undertaken such that the number of colony forming units was much greater than the number of possible unique variants. Biochemistry experiments were performed in biological duplicates or triplicates, wherever applicable, and data were collected in triplicate. The sample sizes were sufficient as they showed differences between samples that were corroborated with orthogonal assays.
Data exclusions	No data relevant to the findings of this study were excluded.
Replication	The MISER technique was performed once overall, and this library was further subdivided into two distinct sub-libraries for sequencing and analysis (Slice 4 and 5; see SI). The MISER library enrichment data presented here is somewhat sparse and only a relative measurement of CRISPRi function. However, the large-scale features of acceptable domain and sub-domain level deletions each contain hundreds of exemplary variants, and furthermore individual representatives were extensively validated by in vivo and biochemical assays. In vivo and in vitro experiments were repeated multiple times to verify reproducibility. Mammalian cell culture experiments were run in triplicates. Comparable results were obtained and are reported as mean +/- SD.
Randomization	Randomization was not relevant to the study as the assays relied on automated readouts from instruments.
Blinding	Blinding was not observed as the assays relied on automated readouts from instruments.

Reporting for specific materials, systems and methods

We require information from authors about some types of materials, experimental systems and methods used in many studies. Here, indicate whether each material, system or method listed is relevant to your study. If you are not sure if a list item applies to your research, read the appropriate section before selecting a response.

Materials & experimental systems

n/a	Involved in the study	
☐	☒	Antibodies
☐	☒	Eukaryotic cell lines
☒	☐	Palaeontology and archaeology
☒	☐	Animals and other organisms
☒	☐	Human research participants
☒	☐	Clinical data
☒	☐	Dual use research of concern

Methods

n/a	Involved in the study	
☒	☐	ChIP-seq
☐	☒	Flow cytometry
☒	☐	MRI-based neuroimaging

Antibodies

Antibodies used	Mouse monoclonal Anti-Flag-M2 (Sigma-Aldrich, #1804, clone M2), HRP-conjugated mouse monoclonal Anti-Beta-Actin (Santa Cruz Biotechnology, #sc-47778 HRP, clone C4), HRP-conjugated sheep Anti-Mouse (GE Healthcare Amersham ECL, #NXA931).
Validation	All antibodies are validated by their respective manufacturers.<br>Mouse monoclonal Anti-Flag-M2 (Sigma-Aldrich, #1804, clone M2): https://www.sigmaaldrich.com/certificates/COFA/F1/F1804/F1804-BULK________SLCD3524__.pdf.<br>HRP-conjugated mouse monoclonal Anti-Beta-Actin (Santa Cruz Biotechnology, #sc-47778 HRP, clone C4): https://datasheets.scbt.com/sc-47778.pdf.<br>HRP-conjugated sheep Anti-Mouse (GE Healthcare Amersham ECL, #NXA931): https://cdn.cytivalifesciences.com/dmm3bwsv3/AssetStream.aspx?mediaformatid=10061&destinationid=10016&assetid=13500.

Eukaryotic cell lines

Policy information about cell lines

Cell line source(s)	U-251 human glioblastoma cells (Sigma-Aldrich, #09063001), HEK293T human kidney cells (293FT; Thermo Fisher Scientific,

	#R70007).
Authentication	U-251 cells were authenticated using short tandem repeat DNA profiling (STR profiling; UC Berkeley Cell Culture/DNA Sequencing facility).
Mycoplasma contamination	HEK293T and U-251 cells were tested for absence of mycoplasma contamination (UC Berkeley Cell Culture facility) by fluorescence microscopy of methanol fixed and Hoechst 33258 (Polysciences, #09460) stained samples. The cell lines tested negative for mycoplasma contamination.
Commonly misidentified lines (See ICLAC register)	No commonly misidentified lines.

Flow Cytometry

Plots

Confirm that:

- [x] The axis labels state the marker and fluorochrome used (e.g. CD4-FITC).
- [x] The axis scales are clearly visible. Include numbers along axes only for bottom left plot of group (a 'group' is an analysis of identical markers).
- [x] All plots are contour plots with outliers or pseudocolor plots.
- [x] A numerical value for number of cells or percentage (with statistics) is provided.

Methodology

Sample preparation	After 8 hours of growth, cultures of E.coli were sampled as follows: 500 µL was centrifuged at 13,000xG for 1 minute, washed once with 1 ml IX PBS, and re-suspended 1:50 in IX PBS for flow cytometry.
Instrument	SH800S Cell Sorter (Sony Biotechnology)
Software	Sony Cell Sorter Software v-2.1.5
Cell population abundance	Cells with repression-competent variants were sorted multiple times to enrich those populations and subsequently isolate high-functioning variants. Abundance of sorted cells are therefore not relevant to the findings of the study.
Gating strategy	For isolating cells with repression-competent Cas9 variants, gates were drawn to capture cells with at least one order of magnitude repression of GFP or RFP.

- [x] Tick this box to confirm that a figure exemplifying the gating strategy is provided in the Supplementary Information.
