# Supplementary material for: Comprehensive deletion landscape of CRISPR-Cas9 identifies minimal RNA-guided DNA-binding modules
Source: Nat Commun. 2021 Sep 27;12:5664. doi: 10.1038/s41467-021-25992-8 (PMC8476515; doi:10.1038/s41467-021-25992-8)
Supplement: Supplementary file 4 — Description of Additional Supplementary Files [file 41467_2021_25992_MOESM4_ESM.pdf]

Title: Supplementary Data 1

Description: Tables of oligonucleotides, plasmid sequences, and DNA fragments used in this study, provided in separate spreadsheets. See Methods section and Supplementary Information for more details.
